# Standardization and validation of assay of selected omega-3 and omega-6 fatty acids from phospholipid fraction of red cell membrane using gas chromatography with flame ionization detector

**DOI:** 10.1186/s40543-021-00287-1

**Published:** 2021-08-06

**Authors:** Ruby Gupta, Savita Dhatwalia, Monica Chaudhry, Dimple Kondal, Aryeh D. Stein, Dorairaj Prabhakaran, Nikhil Tandon, Lakshmy Ramakrishnan, Shweta Khandelwal

**Affiliations:** 1 Public Health Foundation of India, Plot-47, Sector-44, Gurugram, Haryana 122002 India; 2 Centre for Chronic Disease Control, New Delhi, India; 3 Hubert Department of Global Health, Rollins School of Public Health, Emory University, Atlanta, GA USA; 4 All India Institute of Medical Sciences, New Delhi, India

**Keywords:** Docosahexaenoic acid, Gas chromatography, Phospholipid, Validation, Polyunsaturated fatty acids

## Abstract

**Background:**

Docosahexaenoic acid (DHA) is an important structural component of human brain and retina. Evidence exists linking nutritional status of pregnant mothers and cognitive functions of their born infants. The DHANI (Maternal DHA Supplementation and Offspring Neurodevelopment in India) trial was implemented to evaluate the effect of maternal supplementation with DHA during pregnancy and for 6 months following delivery on motor and mental development of infants at 1 and 12 months. We describe here the standardization and validation of an assay for measurement of selected omega-3 and omega-6 fatty acids from the phospholipid fraction of red blood cells to assess their status in mothers at baseline, delivery and 6 months post-delivery and for infants in cord blood and at 1 and 12 months of age. The validated method has been used for the analysis of samples for DHANI.

**Methods:**

Lipids were extracted from a pool of red blood cells, separated using thin layer chromatography. The phospholipid fraction was esterified, and fatty acids were separated by gas chromatography using a flame ionization detector.

**Result:**

The method accuracy for DHA was between 97 - 98% and between 91 - 95% for arachidonic acid (AA) at three different concentrations. The intra-assay and inter-assay coefficient of variation for the fatty acids ranged from 1.19 to 5.7% and 0.78 to 13.0% respectively. Intraclass correlation (ICC), as a measure of reproducibility, ranged between 0.689 and 0.996. A good linearity was observed for all the fatty acids between concentrations of 0.2–4 μg/ml.

**Conclusion:**

The standardized and validated method is suitable for implementation in large epidemiological studies for evaluation of fatty acids and in nutritional trials for assessment of fatty acid content of various lipid classes.

## Introduction

Omega -3 fatty acids play an important role in human health and disease. Many of the long chain polyunsaturated fatty acids (PUFA) of the omega-3 family, DHA (docosahexaenoic acid) in particular, constitute the structural components of phospholipid membrane of the brain and retina and therefore are critical during the developmental phase of the foetus and neurodevelopment of infants in the first 1000 days of their life (Schwarzenberg and Georgieff 2018). The DHANI (Maternal DHA Supplementation and Offspring Neurodevelopment in India), a randomized controlled trial, was implemented to study the effect of pre- and post-natal maternal DHA supplementation on infant motor and mental development, anthropometry and morbidity patterns. The study protocol and primary findings are published elsewhere (Khandelwal et al. 2018, Khandelwal et al. 2020). In short, healthy pregnant mothers (from ≤20 weeks of pregnancy to 6 months postpartum) were supplemented daily with 400 mg of DHA, the neurodevelopment using the Development Assessment Scale for Indian Infants (DASII) was assessed at 6 and 12 months of age, and body size of infants was assessed at birth and 6 and 12 months of age.

To assess the compliance and difference in levels of DHA in pregnant mothers at baseline, delivery and 6 months postpartum, we standardized and validated the basic procedure for the analysis of DHA and other long-chain fatty acids using gas liquid chromatography (GLC or GC). The best way to assess the actual intake by the participant in a trial or any other observational study is to measure fatty acid levels objectively in the body tissue. The supplemented/dietary fatty acids are incorporated into the phospholipid fraction of membranes in a dose-dependent manner (Cao et al. 2006). The primary objective of this validation is to establish a written procedure for process control. It is an essential step in method development and adds to the reliability of data generated in a laboratory setting (Peris-Vicente et al. 2015). We therefore report here the standardization of laboratory procedures for the extraction of lipids from red blood cells (RBC), separation of the phospholipid (PL) fraction by thin layer chromatography (TLC), methylation of PL and separation using GC with flame ionization detection (FID) and assay validation. The fatty acid estimates are semi-quantitative.

## Experimental

### Standards and reagents

Docosahexaenoic acid (DHA, C22:6), arachidonic acid (AA, C20:4), and certified reference material (CRM) of 37-fatty acid methyl ester (FAME) mix were purchased from Supelco. 1,2, Dioleyl sn-glycero-3-phosphoethanolamine and cholesterol as standards for TLC plates were obtained from SIGMA-ALDRICH. Heptadecanoic acid (C17:0) was obtained from RESTEK. All solvents were HPLC grade from MERCK.

### Glass ware

All glassware was meticulously cleaned by rinsing with detergent, washing with hot water and generous rinses with tap water followed by double distilled water. Glassware was drained and dried in an oven at 130 °C overnight or rinsed with methanol and drained (Method 8015B, 1996). All glassware was stored in a dust-free, clean environment. Phthalate esters in plastic ware can interact with transesterification reagents to give mono- and sometimes di-methyl esters; therefore, experiments were conducted only using glassware.

### Instrumentation

The 7890B gas chromatographic system from AGILENT, with a flame ionization detector, and an automated liquid sampler was used. For separation of fatty acids, we dissolved 1 μl of sample in toluene and injected it into the column. The column used for the separation was DB-23 (length 60 m, internal diameter 0.25 mm), 0.25 mm film thickness of a highly polar (50%cyanopropyl)-methyl poly- siloxane stationary phase from AGILENT, Santa Clara, USA. The carrier gas used was nitrogen, with hydrogen and zero-air as make-up gases. All gases were passed through a gas clean filter to trap moisture and other contaminants. The flow rate was 1.0 ml/min. Detector and injector temperatures were 250 °C and 220 °C, respectively. The column temperature was held at 50 °C for 1 min, and then increased at a rate of 25 °C per min until the temperature reached 200 °C, after which the rate was reduced to 4 °C per min until the final temperature of 230 °C was attained; the temperature was held for 7 min. Integration of peaks was performed by Chem-station software (version C.01.07) from AGILENT. The identification of peaks was done by comparing retention times with 37-FAME mixture from Supelco as a reference, as well as spiking of individual fatty acids.

### Samples for the validation study

Blood was collected in EDTA tubes from consenting volunteers and centrifuged to separate out plasma. Red blood cells were pooled and washed thrice with equal volume of saline solution (0.9% NaCl). Multiple aliquots of 200 μl of the pool of RBCs were prepared and stored at − 80 °C for validation analysis. A separate set of pooled RBCs were used for the stability study.

### Ethical approvals

All required ethical approvals for study were obtained from the ethical committee of all the participating institutes: Centre for Chronic Disease Control, All India Institute of Medical Sciences, New Delhi, Public Health Foundation of India, Gurugram and Jawaharlal Nehru Medical College, Belgaum.

### Extraction of lipids

Lipids were extracted from 200 μl red blood cells in glass tubes with Teflon-lined screw caps by adding isopropanol with continuous vortexing. Subsequently, chloroform was added in ratio 11:7, by volume (Rose and Oklander, 1965) with 50 mg/l of butylated hydroxy toluene (BHT) as antioxidant (to prevent autoxidation of PUFA). One hundred microliters of C17:0 was added to every sample as internal standard (0.1 mg/ml). The tube content was shaken in an ultrasonic water bath (20 kHz) for 30 s and equilibrated with one fourth volume of saline solution (0.9% NaCl). The suspension was centrifuged at 3000 × *g* at 4 °C to separate the precipitated proteins and cell fragments. The chloroform layer was separated into amber glass vials and dried under the stream of nitrogen and were stored at − 20 °C till further used for TLC.

### Thin layer chromatography (TLC)

Glass plates (20 × 20 cm) coated with a layer of Silica Gel G (Merck Millipore, Darmstadt, Germany) with 250 μm thickness and 10-12 μm particle size were used for TLC. The plates were washed with a solvent mixture of chloroform: methanol (1: 1, v/v) to remove the impurities and were dried before running the samples. A lipid solution (10 μl) was placed using Hamilton syringe 2 cm above the base of the plate and allowed to dry. A mixture of petroleum ether, diethyl ether and acetic acid (85: 15: 2) as eluent was used to separate the PL fraction from neutral lipids (Gloster and Fletcher 1966). Standards were run simultaneously to confirm the separation. The plate was sprayed with 0.01% (w/v) Primulin in acetone-water (60:40, v/v) and viewed under ultraviolet light (340 nm) for identification of lipid fractions. The phospholipid fraction was marked, scrapped from the plate and re-dissolved in 100 μl of chloroform-methanol (1:1, by volume).

### Fatty acid methyl ester (FAME) preparation

Methyl esters were prepared by adding 1.8 ml of acetyl chloride-methanol (1:20, v/v) to the scrapped PL fraction, vortexed and heated in a water bath at 100 °C for 1 h in glass tubes with Teflon-lined screw caps. The solution was washed with 3.0 ml of 6% cold K_2_CO_3_ to neutralize the excess acid. The esters were extracted in 400 μl of toluene and dried over sodium sulphate (Leepage and Roy 1984). The esters were recovered by drying under a gentle stream of nitrogen, re-dissolved in toluene and subjected to GC.

### Validation characteristics

The proposed method was validated for specificity, precision (repeatability, intermediate precision and reproducibility), accuracy, LOD and LOQ, linearity and stability as per ICH method guidelines (ICH method guidelines 1996).

- *Specificity*: To assess the ability of our analytical method to measure accurately and specifically the analyte in the presence of impurities or degradants that may be expected to be present in the sample, we spiked pooled samples with commercially available individual fatty acid methyl esters of known identity and compared their retention time.
- *Precision evaluation*: To measure the closeness of agreement or degree of scatter in a series of measurements, the same samples were considered at three levels: repeatability, intermediate precision and reproducibility.
  *b. (i). Repeatability* (intra-assay precision): Lipids were extracted from RBC pool in triplicate; the phospholipids were separated by TLC, methylated and run on GC.
  *b.(ii). Intermediate precision*: Expressed as coefficient of variation (CV) of a series of measurements from the sample on different days (inter-assay CV).
  b.(ii).1. For evaluation of inter-assay of the procedure, lipids were extracted from aliquots of pool RBC in triplicate; phospholipid fraction was separated by TLC, extracted from plate, methylated and run on GC on seven different days.
  b.(ii).2. For evaluation of the system, commercially available fatty acid methyl esters of known concentrations were run on GC on seven different days.
  *b.(iii). Reproducibility*: To evaluate the variations due to different equipment, 10% of the randomly selected samples from the Dhani study, blinded for their supplementation status, was also analysed using same process and instrument at the Department of Cardiac Biochemistry, All India Institute of Medical Sciences, New Delhi. The results were evaluated for agreement between the two labs.
- *Method accuracy*: Also termed as trueness, the method accuracy was evaluated from recovery experiments using reference samples containing known amounts of standard substances.
  c. (i). Method accuracy for lipid extraction: Three different concentrations of DHA and AA were added to an RBC pool (in triplicate). Lipids were extracted, esterified and fatty acid assessed by GC. The recovery percentage for each analyte was calculated from the following equation: Recovery % = Observed concentration/Added concentration × 100.
  c.(ii). Method accuracy for esterification: Lipids were spiked with known amounts of DHA and AA (in triplicate), esterified, fatty acid content assessed by GC and recovery calculated.
  c.(iii). Method accuracy for system: Commercially available fatty acid methyl esters of known content (CRM 47885) were run on GC on six different days, and their recovery was calculated.
- *Detection and quantification limit*: Eight successive dilutions of standard FAME (CRM 47885) was prepared and six determinations were made for every concentration. A curve was generated for every parameter under consideration. The slope of the curve was generated, and limit of detection (LOD) and limit of quantification (LOQ) were calculated using the following formula:
  LOD = 3*SE/slope
  LOQ =10*SE/slope
  SE = Standard error
- *Linearity*: A stock solution of 200 μg/ml was diluted to generate five concentrations covering LOQ. Four determinations were done for each concentration. The area under the peaks for each fatty acid was computed for every concentration. A graph was plotted with the area under the peak against concentration. The correlation coefficient for the regression line was computed.
- *Stability*: The European Criteria 2002/657 states that the stability of the analyte in solvent during storage, in matrix during storage and/or sample preparation should be tested.

### Statistical analysis

All the analyses were performed using Stata (version 16.0). To assess the agreement or reliability, intra-class correlation (ICC) coefficient with their 95% confidence interval (95% CI) were computed, and a Bland-Altman plot was constructed for each fatty acid of interest. The ICC indicates the consensus between values from the two laboratories. The ICC value may vary from 0 to 1.00, 0 suggesting no agreement and 1.0 indicating perfect agreement. An ICC value of 0.50 suggests reasonable agreement, 0.61–0.75 suggests good agreement and a value > 0.75 suggests excellent agreement (Landis and Koch, 1977, Fleiss 1981).

## Results and discussion

During our method development, Rose & Oklander (Rose and Oklander, 1965) and Folch (Folch et al. 1957) methods for lipid extraction were evaluated. The percentage of lipids extracted using single extractions was 69–75% by Rose and Oklander’s method and 63–72% by Folch’s method. However, the lipids extracted by Folch method were contaminated with heme and would stain the TLC plate as well as contaminate GC injector liner wool. Therefore, we adopted the Rose and Oklander method for the purpose of the study. The challenge during lipid extraction process was the clumping of red blood cells during the addition of solvent which would inhibit interaction of solvent with the membrane matrix. Therefore, isopropanol was added with continuous vortexing, giving rise to finer precipitate ensuring deep penetration of solvent within the matrix resulting in improved extraction efficiency. The percentage recovery for AA and DHA using single extraction was 46–70% and 54–65% respectively. However, this increased to 72–86% for AA and 68–81% for DHA with two-step extraction. Three-step extraction further improved the percentage of lipids (95% and 98% for AA and DHA respectively).

Separation of lipid components on TLC plates is a function of their polarity. Fresh activation of TLC plates by heating at 110 °C in oven for 30 min before applying the samples for separation was essential for removal of impurities in the silica gel that would otherwise interfere with separation of lipid classes.

As established by Christie (1993), all fatty acids are esterified at approximately the same rate by methanolic hydrogen chloride; therefore, there is minimum loss of specific fatty acids during the esterification process. However, additional precautions were taken during esterification step such as avoiding high temperature for prolonged periods of time and vigorous agitations so as to prevent degradation and loss of short-chain esters. Compounds that may interfere during GC analysis may be formed from the methanol if prolonged heating at high temperature is allowed in the presence of oxygen. Samples containing PUFA requires handling with care and not to be subjected to vigorous conditions like prolonged heating, vortexing and agitation. Reagents that are perfectly satisfactory when used under optimized conditions can be destructive to fatty acids if used carelessly (Christie 1993).

The primary objective of the validation process for the fatty acids analysis was to establish the basis for a written procedure for process control and to demonstrate that the process is suitable for the intended purpose. A summary of the validation parameters undertaken is presented here.

**Fig. 1:**
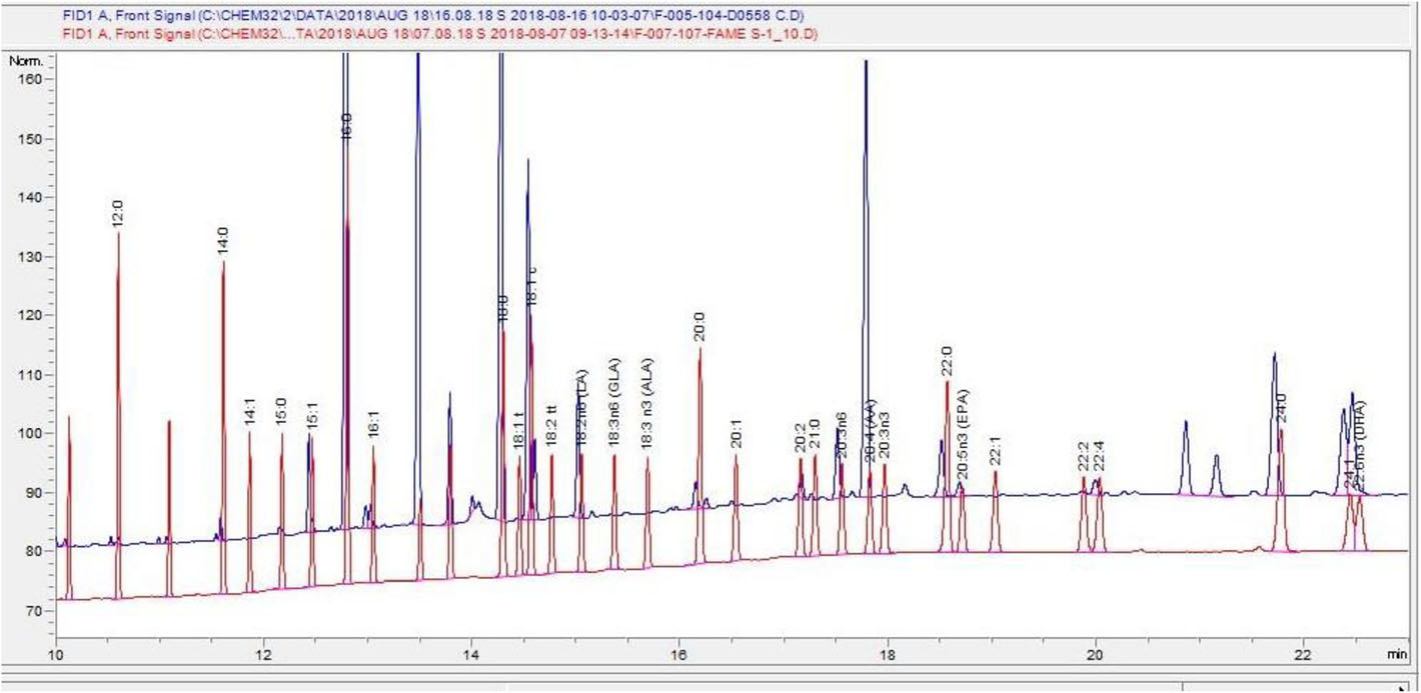
Overlay of FAME mix (red) with an RBC pool (blue)

- *Specificity*: The retention time for fatty acids of interest (omega-3 and omega-6 fatty acids) in a pool sample was confirmed by comparing with commercially available individual as well FAME mixture. An overlay of the pool sample with commercially available FAME mix can be seen in Fig. 1. Thirty fatty acids were identified by comparing their retention time with standard mix. All the peaks were adequately resolved, and there was no interfering peak observed around peaks of our interest. No impurities and carry over were detected when pure solvent was injected before and after the sample run. Matrix associated impurities were eliminated during the use of thin layer chromatography which is known to exclude all non- specific lipid fractions.
- *Precision evaluation:*

- *(i). Repeatability*: Repeatability expresses the precision under the same operating conditions over a short interval of time. Table 1 shows intra-assay precision from six determinations of one RBC pool. As per ICH* criteria, precision should not exceed 15% of the coefficient of variation (CV) except for the LLOQ, where it should not exceed 20% of the CV.
- (ii) *Intermediate precision*: Precision evaluation of the system is shown in Table 1 in terms of inter-assay CV% from commercially available FAME for n-3 and n-6 fatty acids respectively.

**Table. 1:** Intra-assay and inter-assay coefficient of variation from RBC pool and commercially available FAME for n-3 and n-6 fatty acids

Fatty acids	Mean concentration, RBC pool (% of total)	Intra-assay CV % (RBC pool)	Inter-assay CV % (RBC pool)	Concentration, FAME	Inter-assay CV % (FAME)
α-Linolenic acid, C18:3 (n-3)	0.22	5.4	11.07	2	6.25
Eicosapentaenoic acid, C20:5 (n-3)	0.60	5.0	13.0	2	5.31
Docosahexaenoic acid, C22:6 (n-3)	1.92	2.0	2.62	2	3.95
Linoleic acid, C18:2 (n-6)	14.07	1.3	2.33	2	3.15
γ-linolenic acid, C18:3 (n-6)	0.99	5.7	6.04	2	6.87
Arachidonic acid, C20:4 (n-6)	5.96	1.19	0.78	2	9.99

CV, coefficient of variation; FAME, fatty acid methyl ester; RBC, red blood cells

**Fig. 2:**
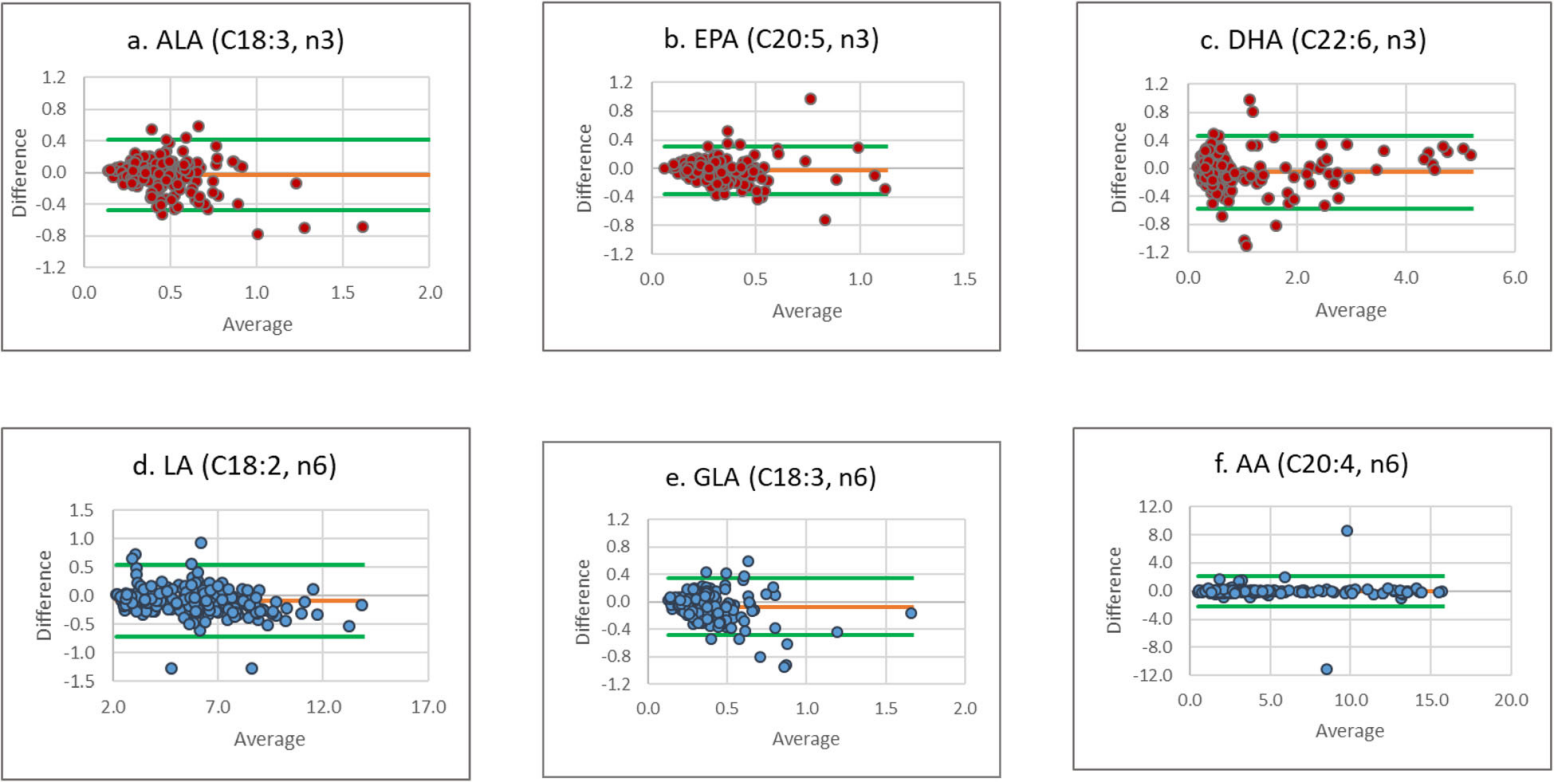
Bland-Altman plot for fatty acids showing average value for the two laboratories versus difference of values between the two laboratories

- *(iii). Reproducibility*: Expressed within-laboratory variations with different analysts, different equipment, etc. The mean for fatty acids analysed at PHFI laboratory was very similar to those analysed at AIIMS laboratory. The mean difference of the two values being very close to zero for all the fatty acids reported. The ICC score shows good agreement between the two laboratories, a minimum score of 0.689 (for gamma-linolenic acid) indicating ‘good agreement’ to 0.996 (for linoleic acid) ‘excellent’ agreement (Table 2). Arachidonic acid and docosahexaenoic acid show an excellent agreement of 0.979 and 0.986 respectively between the two laboratories. The Bland–Altman plot (Fig. 2) shows no systematic bias, and 95% samples are within the ±2SD limits.

**Table. 2:** Agreement of analysis between the two laboratories showing intraclass correlation

Fatty acids	PHFI laboratory (*n* = 180)		AIIMS laboratory (*n* = 180)		Mean difference (95% CI)	ICC (95% CI)
	Mean ± SD	Median (IQR)	Mean ± SD	Median (IQR)		
ALA C18:3(n-3)	0.4549 ± 0.4651	0.3632 (0.258, 0.552)	0.5032 ± 0.5162	0.3774 (0.288, 0.589)	− 0.0260 (− 0.007, − 0.06)	0.832 (0.775–0.875)
EPA C20:5(n-3)	0.3107 ± 0.1792	0.2741 (0.203, 0.363)	0.3390 ± 0.1909	0.2981 (0.213, 0.424)	− 0.0284 (− 0.004, − 0.052)	0.752 (0.667–0.816)
DHA C22:6(n-3)	0.9636 ± 1.0962	0.5027 (0.335, 1.014)	1.0186 ± 1.0658	0.6095 (0.367, 1.101)	− 0.0549 (− 0.016, − 0.093)	0.985 (0.980–0. 989)
LA C18:2(n-6)	5.8225 ± 2.3579	5.6083 (3.742, 7.501)	5.9080 ± 2.4164	5.6816 (3.827, 7.594)	− 0.0855 (− 0.039, -0.131)	0.996 (0.994–0.997)
GLA C18:3(n-6)	0.3509 ± 0.1862	0.3106 (0.225, 0.423)	0.4192 ± 0.2421	0.3653 (0.265, 0.505)	− 0.0683 (− 0.037, − 0.099)	0.689 (0.582–0.768)
AA C20:4(n-6)	4.6899 ± 3.7776	3.2405 (2.249, 5.406)	4.7023 ± 3.8154	3.1651 (2.267, 5.418)	− 0.0124 (0.148, − 0.173)	0.979 (0.971–0.984)

PHFI, Public Health Foundation of India; AIIMS, All India Institute of Medical Sciences; ICC, intraclass correlation coefficient; CI, confidence interval; IQR, interquartile range; ALA, alpha linolenic acid; EPA, eicosapentaenoic acid; DHA, docosahexaenoic acid; LA, linoleic acid; GLA, gamma-linolenic acid; AA, arachidonic acid

iii. *Method accuracy*: was expressed as percent recovery of the analyte. The accuracy with lipid extraction in our study varied from 91 to 95% and 97 to 98% for AA and DHA respectively (Table 3) as we move from low to higher concentration (2.5 μg/ml, 12.5 μg/ml and 25 μg/ml for as 10%, 50% and 100% respectively of AA and DHA). However, the recovery percentage with esterification was close to 94% and 97%, whereas for the system, it was 98% and 96% for AA and DHA respectively (Table 4).

**Table. 3:** Accuracy results for lipid extraction

Fatty acids		Mean % recovery (***n*** = 9)	RSD %
Arachidonic acid, C20:4	10%	91.8	5.84
	50%	95.2	1.03
	100%	95.4	1.44
Docosahexaenoic acid, C22:6	10%	97.7	2.70
	50%	98.6	2.64
	100%	97.9	2.72

RSD, relative standard deviation

**Table. 4:** Accuracy results for esterification and system

Fatty acids	Esterification accuracy		System accuracy	
	Mean % recovery	RSD % (***n*** = 9)	Mean % recovery	RSD % (***n*** = 9)
α-Linolenic acid, C18:3 (n-3)	–	–	110.19	0.93
Eicosapentaenoic acid, C20:5 (n-3)	–	–	90.49	0.74
Docosahexaenoic acid, C22:6 (n-3)	97.56	4.88	96.04	1.89
Linoleic acid, C18:2 (n-6)	–	–	105.15	0.27
γ-linolenic acid, C18:3 (n-6)	–	–	104.36	0.78
Arachidonic acid, C20:4 (n-6)	94.51	0.29	98.10	0.46

*RSD* relative standard deviation

iv. *Detection and quantification limit*: LOD and LOQ were estimated from the FAME mix standard solution (Table 5). The curves generated followed a linear behaviour, ‘*r*’ ranging from 0.991 for EPA to 0.997 for AA.

**Table. 5:** Detection (LOD) and quantification limits (LOQ)

Fatty acids	LOD (μg/ml)	LOQ (μg/ml)	Correlation coefficient (***r***)
α-linolenic acid, C18:3 (n-3)	0.118	0.394	0.992
Eicosapentaenoic acid, C20:5 (n-3)	0.085	0.283	0.991
Docosahexaenoic acid, C22:6 (n-3)	0.080	0.263	0.996
Linoleic acid, C18:2 (n-6)	0.125	0.415	0.992
γ-linolenic acid, C18:3 (n-6)	0.110	0.368	0.992
Arachidonic acid, C20:4 (n-6)	0.086	0.286	0.997

LOD, limit of detection; LOQ, limit of quantification

e. *Linearity*: The correlation coefficient ranges from 0.996 for docosahexaenoic acid (C22:6) to 0.997 for linoleic acid (C18:2). The details are summarized in Table 6.

**Table. 6:** Linearity with sample concentration

Fatty acids	Concentration range (μg/ml)	Regression equation ***Y*** = ax + b	Correlation coefficient “***r***”
α-Linolenic acid, C18:3 (n-3)	0.2–4	48.528x − 0.4636	0.9971
Eicosapentaenoic acid, C20:5 (n-3)	0.2–4	49.153x − 0.6419	0.9971
Docosahexaenoic acid, C22:6 (n-3)	0.2–4	45.859x − 0.511	0.9965
Linoleic acid, C18:2 (n-6)	0.2–4	49.244x − 0.252	0.9972
γ-linolenic acid, C18:3 (n-6)	0.2–4	48.424x − 0.3464	0.9971
Arachidonic acid, C20:4 (n-6)	0.2–4	47.8x − 0.5545	0.9970

f. *Stability*: As DHANI was being conducted with pregnant females, samples were collected at the time of delivery, which could happen at odd hours and there may be delay in processing. Therefore, we assessed stability of blood sample (stored at 4 °C) and processing delays of 6 h and 12 h (Table 7).

**Table. 7:** Stability of fatty acids due to delay in processing

Fatty acids	% peak composition (absolute difference)		
	BL	6 h	12 h
α-Linolenic acid, C18:3 (n-3)	0.18	0.18 (0.00)	0.17 (0.01)
Eicosapentaenoic acid, C20:5 (n-3)	0.28	0.29 (− 0.01)	0.22 (0.06)
Docosahexaenoic acid, C22:6 (n-3)	1.90	1.88 (0.02)	1.86 (0.04)
Linoleic acid, C18:2 (n-6)	13.25	13.67 (− 0.42)	13.96 (− 0.71)
γ-linolenic acid, C18:3 (n-6)	0.26	0.21 (0.04)	0.14 (0.11)
Arachidonic acid, C20:4 (n-6)	11.28	11.23 (0.05)	11.29 (0.01)

BL, baseline

In addition, there were possibilities of delay in analysis of samples after esterification; therefore, stability of esters was evaluated at different temperature conditions (room temperature and 4–8 °C) for 3 days, 5 days and 8 days. Omega-3 fatty acids tend to degrade faster at room temperature (Table 8). However, methyl esters have been shown to be stable for a month when stored at − 20 °C (Supelco Safety Data sheet 2021).

**Table. 8:** Stability results after storage of methyl esters at different temperatures

	**% peak composition (absolute difference)**							
**Fatty acids**	**RT**				**4 – 8**^**o**^			
	**Day 1**	**Day 3**	**Day 5**	**Day 8**	**Day 1**	**Day 3**	**Day 5**	**Day 8**
ALA, C 18:3, (n-3)	0.46	0.44 (0.02)	0.33 (0.13)	0.27 (0.19)	0.43	0.41 (0.02)	0.34 (0.09)	0.26 (0.17)
EPA, C 20:5, (n-3)	0.46	0.35 (0.11)	0.24 (0.22)	0.11 (0.35)	0.26	0.17 (0.08)	0.20 (0.06)	0.18 (0.08)
DHA, C 22:6, (n-3)	1.22	1.10 (0.12)	1.18 (0.04)	1.03 (0.19)	1.12	1.16 (-0.04)	1.10 (0.01)	1.05 (0.07)
LA, C 18:2, (n-6)	5.66	5.49 (0.17)	5.78 (-0.12)	5.80 (-0.14)	5.65	5.64 (0.01)	5.74 (-0.10)	5.81 (-0.17)
GLA, C 18:3, (n-6)	0.41	0.46 (-0.04)	0.35 (0.06)	0.12 (0.29)	0.43	0.47 (-0.04)	0.28 (0.15)	0.23 (0.20)
AA, C 20:4, (n-6)	6.12	6.01 (0.11)	6.10 (0.02)	6.23 (-0.11)	6.06	6.03 (0.02)	6.17 (-0.12)	6.29 (-0.24)

*RT* room temperature, *ALA* alpha linolenic acid, *EPA* eicosapentaenoic acid, *DHA* docosahexaenoic acid, *LA* linoleic acid, *GLA* gamma-linolenic acid, *AA* arachidonic acid

The method described above is specific, accurate and precise and can be standardized with short training. The validated method has been used for the analysis of omega-3 and omega-6 fatty acids in DHANI study (Khandelwal et al., 2020, Khandelwal et al. 2021). The method can also be used for evaluation of fatty acids in large nutritional trials and as biomarker assessment for large epidemiological studies.

## Conclusion

A method for extraction of lipids from red blood cells, their separation using TLC and analysis of fatty acids using GC-FID was validated. Thirty fatty acids were identified using a program with a total run time of 22 min; six of them were validated for the study. The method is free from interference from the diluents used for extraction and separation of PL fraction and demonstrates specificity for the studied fatty acids. The method as described above is specific, accurate, precise and robust for the analysis of DHA and AA from PL fraction of RBC membrane. The method has been used for assessment of fatty acids in DHANI and is suitable for nutritional trials as well as large epidemiological studies assessing fatty acids as biomarker for various health conditions.

## Data Availability

Not applicable

## Abbreviations

PUFA: Polyunsaturated fatty acids
DHA: Docosahexaenoic acid
GC: Gas chromatography
FID: Flame-ionization detector
PL: Phospholipid
LA: Linoleic acid
GLA: Gamma-linolenic acid
AA: Arachidonic acid
ALA: Alpha linolenic acid
EPA: Eicosapentaenoic acid
DHA: Docosahexaenoic acid
LOD: Limit of detection
LOQ: Limit of quantification
